# Impact of urban agriculture on malaria vectors in Accra, Ghana

**DOI:** 10.1186/1475-2875-7-151

**Published:** 2008-08-04

**Authors:** Eveline Klinkenberg, PJ McCall, Michael D Wilson, Felix P Amerasinghe, Martin J Donnelly

**Affiliations:** 1 International Water Management Institute (IWMI), West Africa Office, Ghana; 2 Liverpool School of Tropical Medicine, Liverpool, UK; 3 Noguchi Memorial Institute for Medical Research, Legon, Ghana; 4 IWMI Headquarters, Colombo, Sri Lanka; 5 Current address: KNCV Tuberculosis Foundation, Parkstraat 17, 2514 JD, The Hague, The Netherlands

## Abstract

To investigate the impact of urban agriculture on malaria transmission risk in urban Accra larval and adult stage mosquito surveys, were performed. Local transmission was implicated as *Anopheles spp. *were found breeding and infected *Anophele*s mosquitoes were found resting in houses in the study sites. The predominant *Anopheles *species was *Anopheles gambiae s.s.*. The relative proportion of molecular forms within a subset of specimens was 86% S-form and 14% M-form. *Anopheles spp. *and *Culex quinquefasciatus *outdoor biting rates were respectively three and four times higher in areas around agricultural sites (UA) than in areas far from agriculture (U). The annual Entomological Inoculation Rate (EIR), the number of infectious bites received per individual per year, was 19.2 and 6.6 in UA and U sites, respectively. Breeding sites were highly transitory in nature, which poses a challenge for larval control in this setting. The data also suggest that the epidemiological importance of urban agricultural areas may be the provision of resting sites for adults rather than an increased number of larval habitats. Host-seeking activity peaked between 2–3 am, indicating that insecticide-treated bednets should be an effective control method.

## Background

There has been a resurgence of interest in the problem of urban malaria in sub-Saharan Africa in recent years [1-5]. Urban malaria is likely to increase in importance as rapid urbanization will result in the majority of Africa's population living in cities in the near future [6]. It is commonly assumed that urbanization leads to a decrease in malaria prevalence because it results in fewer *Anopheles *breeding sites, reduced biting rates due to the higher ratio of humans to mosquitoes [2], better access to treatment and better (mosquito-proof) housing (overview in [7]). However, there is a concern that areas with rapid, unplanned urbanization, typically associated with low income, poor education, poor health care and poor housing/sanitation, may not experience such marked decreases in malaria transmission[1].

Urban malaria epidemiology will pose different challenges to those in rural areas [2]. One concern is that urban agriculture, promoted to increase food security and alleviate poverty [8] might, especially when irrigated, increase the urban malaria risk by creating breeding sites for the *Anopheles *vector [9-12]. Several studies have recorded breeding of *Anopheles *in urban agricultural sites, but few studies have investigated the impact of urban agriculture on entomological and epidemiological indicators. In urban Bouaké, Côte d'Ivoire, higher vector densities were found in rice growing areas than market garden areas, although sporozoite infection rates were lower and the impact on malaria epidemiology was not quantified [13,14]. Robert *et al *[15]suggested that the market garden wells in urban Dakar, Senegal, might not be the most important mosquito breeding grounds as the presence of larvae in the wells did not coincide with the vector density peaks. Matthys *et al *[16] found that urban farming created additional breeding sites for anophelines in the city environment and that malaria risk was affected by the type of farming present. However, in a recent study in two cities in Kenya, Keating *et al *[12] found no association between household level farming and vector breeding sites. Entomological studies in Kumasi, Ghana, found higher *Anopheles *biting rates and significantly more reported malaria cases in urban areas with agriculture compared to urban areas without agriculture [9], though later epidemiological studies indicated that living near urban agriculture was not associated with malaria parasitaemia in young children in Kumasi [17].

Variously, findings of these earlier studies suggested that urban agricultural areas, while supporting *Anopheles *breeding, do not necessarily result in a detectable increase in malaria risk.

Entomological and epidemiological studies were performed in urban Accra, Ghana, to assess the impact of urban agriculture on malaria transmission risk. Epidemiological surveys indicated that in urban Accra, malaria prevalence was significantly higher in children in communities near urban agriculture (UA) than in children in communities far from it [10,17,18]. However, only in some communities was there a significant inverse relationship between distance to agriculture and malaria prevalence. Also there were communities far from agriculture with very high malaria prevalence, indicating that there are likely to be other important risk factors for urban malaria.

Data from a series of entomological studies carried out in urban Ghana are presented and discussed with respect to earlier epidemiological studies. Mosquito breeding and densities in an urban setting were documented and *Plasmodium *infected mosquitoes were identified.

The insecticide susceptibility status of *Anopheles sp. *is discussed because in addition to providing breeding sites, urban agriculture and the associated extensive use of pesticides, could select for resistance to the pesticides used in public health [19-21].

## Methods

Entomological surveys were carried out in the same communities in Accra as the epidemiological surveys described previously [10]. Communities were categorized by their proximity to sites of agriculture as either an urban agricultural community (UA) or if more distant, an urban community (U). Details of the community selection and categorization procedures were given in Klinkenberg *et al *[10]. The study was approved by the ethical review committees of the Liverpool School of Tropical Medicine and the Noguchi Memorial Institute for Medical Research, University of Ghana.

### Adult collections

**Figure 1:**
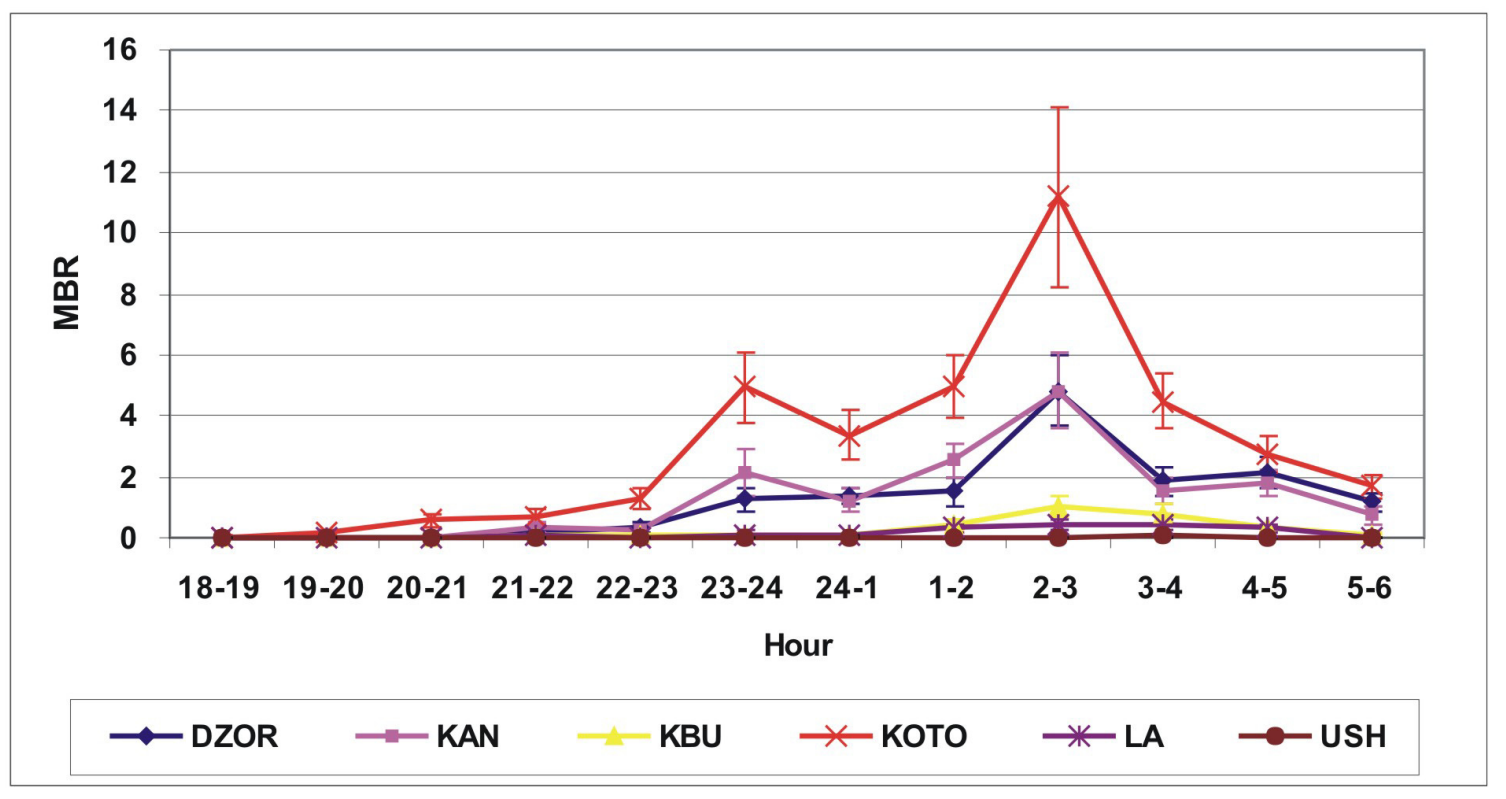
Hourly man biting rate (with standard error) for *Anopheles gambiae s.l. *(average of eight rounds) in selected communities in Accra.

From the 8th September – 19th December 2003, eight rounds of human landing catches (HLCs) were carried out fortnightly in six selected communities in Accra to estimate man biting rates, mosquito parity rates and nocturnal biting activity. Human landing catches were carried out in three UA sites (Kotobabi, Dzorwulu and Korle Bu) and three U sites (Kaneshie, La and Ushertown) (see Figure 1 in reference [10]). Two different communities were surveyed per night (one UA and one U). Two fixed sampling locations, a few houses apart, were used within each community, and two pairs of catchers were based at each sampling location. Catchers were selected from the local community to facilitate acceptance from residents. Informed consent was obtained from each catcher and malaria prophylaxis was provided. All collections were performed outdoors. Mosquitoes were caught from 6 pm to 6 am and hourly collections were stored separately. Mosquitoes were caught by a tube when landing on the leg and transferred to a paper cup with a netting lid following methods described in Service [22]. The catchers were trained to collect landing mosquitoes prior to blood feeding, to minimise the risk of malaria transmission. Catches were transported back to the laboratory in the morning for identification and processing.

In addition to the human landing catches, monthly rounds of pyrethrum knockdown catches (PKD) were planned in 11 communities in Accra and started in October 2003 (all communities of the epidemiological survey [12] except Cantonments, because of the low number of residential houses around the UA zone). Due to the low numbers of mosquitoes caught in the first three rounds, more catches were not conducted (see results). In the selected study communities, in 15 houses in different parts of the community, PKDs were performed as described by Service [22]. Briefly, white sheets were spread over the floor of the room after which windows and doors were closed and rooms were sprayed using locally available aerosol insecticides ('*Mortein*' brand: Bioallethrin 0.12%, Bioresmethrin 0.08%, Tetramethrin 0.38%, solvent and propellant 99.42%). After 15 minutes all mosquitoes were collected from the sheets, transferred into paper cups with a netting lid and transported back to the laboratory for identification. Bloodmeals from fed *Anopheles *mosquitoes were conserved by squashing the abdomen on filter paper and stored over silica gel. For each house, the number of people that slept in the PKD room the previous night was noted and house characteristics such as presence of ceiling, type of wall, and socio-economic score (as described previously [10]) were noted.

### Larval collections

To investigate the range of sites where *Anophele*s could be found breeding in urban areas, a larval survey was carried out in five residential areas in Accra and in the three main urban agricultural sites between September 2003 and March 2004. Breeding sites were located in both UA and U areas by searching through the area to identify and investigate water bodies with the potential to harbour mosquito larvae. Larvae were collected by the dipping method [22]. Habitats were characterised using a standard format for each site, recording presence of vegetation (in/around site), presence of predators (*i.e. *dragonfly, water beetle, water scorpion *etc*.), water quality (pH, Electrical conductivity (EC), foul smell, clear or turbid), light conditions (sunlit or shaded), substratum type, and whether the site was manmade or natural. The pH and electrical conductivity (EC) were measured using a portable pH/EC meter (WTW, Germany pH/cond 340i).

In addition to the inventory of the range of breeding sites as described above, specific surveys were carried out in the UA areas to find out the pattern of breeding in the wells used for irrigation. This was done at the three main agricultural sites in Accra where wells were the most common irrigation structure, Dzorwulu Farm, Kotobabi Farm and Korle Bu Farm. In Korle Bu Farm, all wells were filled by drain water (100%), at Kotobabi Farm nearly all were filled by piped water (95%), while at Dzorwulu Farm, part was filled by drain water (55%), part by piped water (40%) and some by a mixture of piped and drain water (5%). Between December 2003 and May 2004 three inventories were made of the wells in all three areas to assess the percentage of wells containing mosquitoes. This was done by surveying the surface of each well using a small fishing net after which the net was emptied in a white tray to investigate if mosquito larvae or other fauna were present. For each well it was noted if the well was positive or negative for mosquito larvae, if positive, number and type of larvae was noted, a distinction was made between anophelines and other culicines. Habitat characteristics were recorded as described above.

### Mosquito identification and processing

All anophelines were identified to species level, culicines to genus level, e.g. *Culex, Aedes, Mansonia etc*. *Anopheles *larvae from the larval collections were reared in the laboratory to adult stage for easier identification. All adult *Anopheles *were identified to species level following the key of Gillies and de Meillon [23]. A sub sample of the *Anopheles gambiae s.l*. caught in human landing catch was identified to species level by polymerase chain reaction (PCR) following Scott *et al *[24]. All *A. gambiae s.s. *of this sub sample were identified further to molecular form following Fanello *et al *[25].

For the subsample of *A. gambiae s.l. *for PCR, DNA was extracted from the abdomen and legs using a modified version of the Livak protocol [26] for subsequent species identification by PCR. Heads and thoraces of all *Anopheles *caught during the human landing catch (including the trial round) were processed by sandwich ELISA after Wirtz *et al *[27] to assess sporozoite infection level.

### Insecticide resistance testing

*Anopheles spp. *collected either as larvae and raised to adulthood or adults collected by light trap in 2004/2005 were tested for permethrin susceptibility status using the standard WHO protocols [28]. Up to 20 mosquitoes were exposed for one hour in a WHO tube test containing insecticide-treated paper (0.75% permethrin) and allowed to recover for 23 hours after which mortality was recorded. In addition, cone tests were performed on deltamethrin-treated nets (PermaNet^®^, Vestergaard-Frandsen). Up to 10 mosquitoes were put in a cone on the net for an exposure time of one hour after which they were transferred to paper cups and mortality was assessed 23 hours later.

### Statistical analysis

Man biting rates (+1) estimated from human landing catch and PKD collections were log transformed to normalize the data and analysed by *t*-tests or, if they could not be normalized, by Mann-Whitney U tests. Differences between geometric means were calculated by a two sample *t*-test using the general linear model in SPSS (version 12.0.1). For the larval study, habitat characteristics were linked to presence of mosquitoes by *t*-test for difference between means.

## Results

### Species composition and man biting rates (MBR)

A total of 21,801 mosquitoes were collected by human landing catch in 192 man nights; species composition is given in Table 1. The majority (92%) were *Culex spp*. and the remainder were *Anopheles spp*, over 99% of which were *Anopheles gambiae s.l. *The six *Anopheles coustanii *were all caught in the same night at one site (Dzorwulu). A subset of 112 of the *A. gambiae s.l. *caught was successfully identified by PCR and all specimens were *A. gambiae s.s. *of which 96 (85.7%) were S-form and 16 (14.3%) were M-form. The results from the pyrethrum knockdown collections showed a similar species composition (Table 1), with the majority being *Culex spp.*

**Table 1:** Species composition of mosquitoes collected in human landing collections and pyrethrum knockdown catches in Accra.

**Method**	**Human landing ** **collection**	**Pyrethrum knockdown ** **collection**
**No. of rounds**	8	3
**No. man nights or houses**	192	408
**Total mosquitoes caught**	21,801	4,135
**No. of *Culex spp.***	20,100 (91.8%)	3,915 (94.7%)
**No. of *Anopheles spp.***	1,648 (7.6%)	153 (3.7%)
*A. gambiae s.l.*	1,642 (99.6%)	146 (95.4%)
*A. funestus*	0 (0%)	7 (4.6%)
*A. coustani*	6 (0.4%)	0 (0%)
**No. of *Aedes spp.***	111 (0.5%)	67 (1.6%)
**No. of *Mansonia spp.***	32 (0.1%)	0 (0%)

Daily man biting rates (MBR) estimated from the human landing catch and pyrethrum knockdown collections were markedly different, pyrethrum knockdown collection MBRs were much lower (Table 2). The geometric mean of the daily biting rates obtained by human landing catch was about three times higher in UA compared to U communities for *A. gambiae s.l*. and four times higher for *Culex spp *(Table 2). The mean biting rate showed marked variation between communities (Table 3). The human population received from five to fifty-five times as many *Culex *as *Anopheles spp *bites.

**Table 2:** Mean nightly man biting rate, with 95% confidence intervals, from human landing collections and pyrethrum knockdown collections for urban communities with and without agriculture.

	**Human landing catch**		**Pyrethrum knockdown collection**	
	***A. gambiae***	***Culex***	***A. gambiae***	***Culex***

**Urban Agriculture**	8.1 (5.1–13.0)	161.8 (132.1–198.1)	0.43 (0.4–0.5)	1.1 (0.9–1.3)
**Urban**	2.8 (1.8–4.3)	41.0 (27.8–60.4)	0.37 (0.3–0.4)	0.7 (0.6–0.8)
**All communities**	4.7 (3.3–6.7)	81.4 (60.8–109.1)	0.40 (0.34–0.42)	0.9 (0.8–1.0)

Data were analysed based upon a log_10_(n+1) transformation.

**Table 3:** Mean nightly man biting rate, with 95% confidence intervals, from human landing collections for the six study communities.

**Community**	**Type**	**MBR ** ***Culex spp.***	**MBR ** ***A. gambiae s.l.***	**EIR ** ***A. gambiae s.l.***
**Dzorwulu**	UA	234.4 (180.0–305.2)	11.7 (7.9–17.3)	27.82
**Korle Bu**	UA	141.4 (99.2–201.8)	2.6 (1.5–4.4)	6.07
**Kotobabi**	UA	127.8 (94.4–173.1)	18.8 (9.4–37.7)	44.72
**Kaneshie**	U	48.5 (26.9–87.3)	10.6 (6.0–18.9)	25.15
**La**	U	38.4 (19.2–76.8)	2.1 (1.4–3.1)	4.94
**Ushertown**	U	36.9 (16.6–81.9)	1.1 (1.0–1.2)	2.57

The annual entomological inoculation rates is estimated from the mean nightly man biting rate multiplied by 365 and the sporozoite rate 0.65%

MBR = man biting rate; EIR = annual entomological inoculation rate; UA = community near urban agriculture; U = community far from urban agriculture. Data were analysed based upon a log_10_(n+1) transformation.

### Sporozoite rate and EIR

A total of 11/1,672 (0.65%) *Anopheles *from the human landing catch were circumsporozoite protein positive, and all were *A. gambiae s.l. *Seven of these were from Kotobabi, two from Korle Bu, one from Kaneshie and one from Dzorwulu. Combining the sporozoite rate with the MBR from the human landing catches, annual EIRs of 19.2 and 6.6 were calculated for UA and U communities respectively. The estimated EIR for each community is shown in Table 3 although it should be noted that the number of circumsporozoite protein positive mosquitoes were few.

### Nocturnal biting pattern

**Figure 2:**
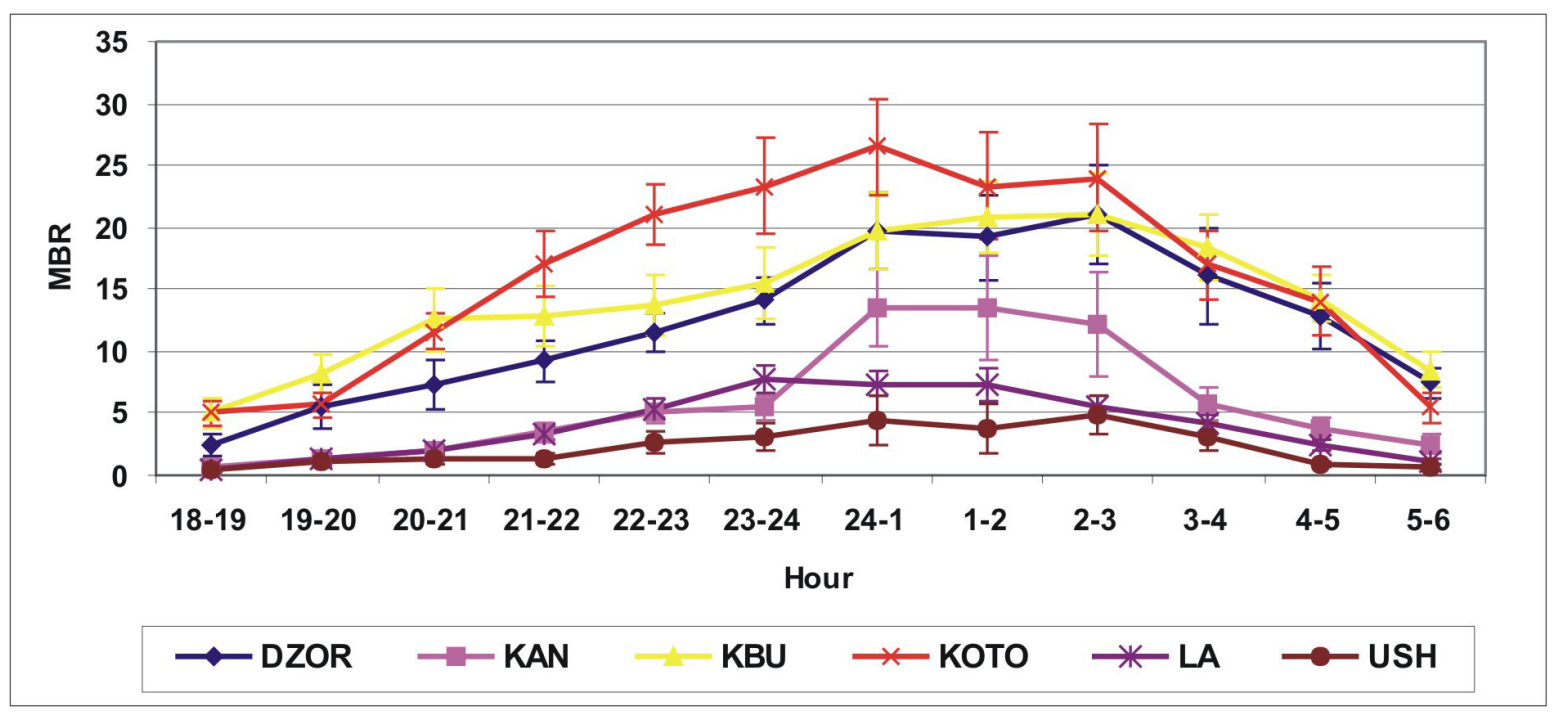
Hourly man biting rate (with standard error) for *Culex spp. *(average of eight rounds) in selected.

*Anopheles gambiae s.l. *nocturnal biting peaked at 2.00–3.00 hrs with the majority biting between 23.00 and 5.00 hrs (Figure 1). *Culex spp. *biting increased after dusk but did not show such a marked peak (Figure 2).

### Larval surveys

*Anophele*s *spp. *were found breeding in both agricultural and residential areas. Larval breeding sites found in the urban residential communities included broken water pipes, pools at construction sites, areas with 'up-welling' water, poorly maintained drains filled with rain water and garbage and rain pools or flooded areas in low lying areas after heavy rains. The first four were typical urban sites although the latter could also be found in rural areas. Open drains were common breeding sites for *Culex spp *mosquitoes.

In the agricultural areas, *Anophele*s were found breeding mainly in the wells used for irrigation, although some could be found in foot prints and seepage areas. A total of 490 wells were examined on thirteen different dates between September 2003 and May 2004. Overall, 6% of the wells were positive for *Anopheles *and 11% for *Culex spp. *There were significant differences in number of positive wells, for both *Anopheles *and *Culex*, between the three farm sites linked to water quality (Table 4). In Dzorwulu and Kotobabi, where wells were filled with drain or piped water, there were significant more wells positive for *Anopheles *that were filled with piped water than wells filled with drain water (*P *= 0.017 and *P *= 0.046 Pearson Chi-square for Dzorwulu and Kotobabi respectively). Average EC was significantly higher in wells where *Culex *larvae were present (*P *< 0.001) and there were significantly more *Culex *larvae in water with a foul smell (*P *< 0.001) while significantly more *Anophele*s larvae were present in non foul smelling water (*P *= 0.017).

**Table 4:** Overview of agricultural wells surveyed, with number positive for Anopheles and Culex spp. and average pH and EC at the three main farm areas in Accra.

**Farm area**	**No. of Wells**	**No. positive for ** ***Anopheles***	**No. positive for ** ***Culex***	**pH (SEM)**	**EC (SEM)**
Dzorwulu	370	23 (6.2%)	16 (4.3%)	7.2 (0.09)	726 (68.2)
Kotobabi	59	7 (11.9%)	1 (1.7%)	6.8 (0.07)	519 (61.3)
Korlebu	61	0 (0%)	36 (59.0%)	7.2 (0.08)	1709(55.3)

EC = Electrical conductivity; SEM = standard error of mean.

### Bioassays to determine insecticide susceptibility status

Between July and December 2004, a total of 305 adult *A. gambiae s.l. *were tested for resistance to permethrin (1 hr. exposure to 0.75% permethrin, WHO paper): 157 (51.5%) were resistant, *i.e. *still alive 23 hrs post a one hour exposure. Resistance was calculated at 55% (106/194) in females and 46% (51/111) in males. There was no significant difference in resistance between mosquitoes from UA and U areas (*P *= 0.31). The cone tests on the insecticide treated nets showed similar results. Of the 119 lab-reared adult *A. gambiae s.l. *that were tested on the insecticide-treated nets (PermaNet^®^, Vestergaard-Frandsen) in five series between July and September 2004, 77 (64.7%) were resistant, *i.e. *still alive 23 hr after recovery from one hour exposure.

## Discussion

The data presented show malaria vectors breeding and biting in urban areas in Accra and the presence of infective mosquitoes demonstrates that malaria transmission occurs within households in these communities. The importance of local transmission is reinforced by associated epidemiological studies, where no association was found between travel outside Accra and presence of malaria parasites in local communities [10]. Clearly, significant levels of malaria are transmitted by local vector populations. The importance of urban agriculture in sustaining such levels is demonstrated by the higher EIR recorded from localities closer to cultivated sites than in those further away.

This study showed that biting rates were markedly heterogeneous across the urban landscape. Similar heterogeneities in malaria prevalence have also been observed in human populations in both Accra and Kumasi, Ghana [10,17,18]. The differences in malaria prevalence can be remarkably stable overtime [17,18] and suggests that in a resource limited setting that focal vector control for urban areas may be appropriate [2,5,7,29]. Outdoor biting activity, which is likely to reflect indoor biting activity, peaked around 2.00 – 3.00 *a.m.*, suggesting that ITNs are likely to be an effective malaria control strategy in this setting. The low numbers of mosquitoes obtained by indoor knockdown catches compared to outdoor landing catches suggests that indoor residual spraying (IRS) may be less effective although this requires further investigation for confirmation. The observed high resistance levels are worrying and could jeopardize the success of a bednet or other control programme dependent on the insecticides used. A recent paper from Benin, West Africa, reported that in an area close to the capital Cotonou, where the vectors are known to display pyrethroid resistance, mosquito feeding was uninhibited by ITNs and mosquito mortality rates were only 30% in an experimental setting [29]. Development of resistance in West Africa has been reported by others [30-34] but further studies are needed, particularly as ITNs ares currently being scaled-up to national levels in several countries in West Africa.

The larval surveys revealed breeding of *A. gambiae s.l. *both in the agricultural sites as well as the normal urban housing areas and although larvae were found in irrigation wells, on average, only 6% of these wells were found to harbour *Anophele*s larvae. This could make targeted larval control difficult because as in rural areas, other breeding sites, often transitory were found in the residential areas. In Dar Es Salaam, Tanzania, for example, larval control implementation at community level was affected by a similar problem [35].

Outdoor man biting rates were significantly higher in UA communities than in U communities, as found in other cities in West Africa [9,13,14]. Interestingly, indoor *Anopheles spp. *man biting rates obtained from pyrethrum spray catches were very low, at approximately 1 per person per night, and did not differ between UA and U. This could indicate that *Anopheles spp*. prefer resting outdoors in this urban setting and that the epidemiological importance of urban agricultural areas may be in providing resting sites for mosquitoes. Robert *et al *[15] earlier suggested that the importance of UA may not solely be the provision of breeding sites as in their study of agricultural wells in Dakar, Senegal, they found that adult density patterns did not follow larval breeding patterns in the wells. Additional behavioural studies are required to characterise the feeding and resting behaviour of these populations

In addition, urban agriculture may promote the rapid development of insecticide resistance in urban areas as urban agriculture, apart from being dependent on a continuous supply of water and nutrients, also uses high inputs of pesticides in intensive crop cultivation [36]. High pesticide use in farming could favour selection for resistance to pesticides used in vector control [19,20]. Moreover, high use of mosquito coils and aerosols in urban areas could add to this selection pressure (*e.g. *35.7% of households used coils daily and 28.8% used aerosols at a weekly basis in Accra, data this study). Although the resistance test carried out in this study did not show a significant difference between UA and U areas, additional studies are needed to investigate this further. Other researchers have also found high resistance levels in mosquitoes from urban areas and sites with intensive agriculture [32].

Although *A. gambiae s.l. *is known to prefer relatively clean water for breeding they were also found breeding in more polluted *e.g. *foul smelling sites with floating garbage. The breeding of *Anophele*s *spp. *in polluted water in urban areas has been reported previously [12,37-39] and could point to a local adaptation or phenotypic plasticity. There are no published results on possible adaptations of *A. gambiae *to more polluted sites but a small common garden experiment carried out in Kumasi [40], wherein urban *A. gambiae s.s*. mosquitoes were reared in rural (clean) water and rural *Anophele*s in urban (polluted) water, and vice versa, indicated that that median time to pupation was longer for rural larvae in urban water. The potential for *A. gambiae s.l. *to adapt to breeding in polluted water is clearly an important area that needs further study as this could have important implications for urban malaria epidemiology.

In urban malaria control there is a clear role for municipalities and public works departments [5]. Proper construction of drains and sewage systems would reduce the amount of open drains proliferating high nuisance *Culex spp *breeding at present. The larval inventory revealed that broken pipes and pools formed at construction sites were major *Anopheles *larval breeding sites in the urban housing areas and this is clearly related to urban expansion outpacing infrastructure development. This was also stressed by Keating *et al*, who found the majority of breeding sites in unplanned, poorly-drained areas in urban Kenya.

The overall EIR calculated from the human landing catches in central Accra was 11.2 ranging from 2.6 – 44.7 infective bites per person per year in the different communities, with an EIR of 19.2 for UA and 6.6 for U areas. These values are comparable to the mean annual EIRs of 7.1 in the city centres, 45.8 in periurban areas, and 167.7 in rural areas reported by Robert *et al. *[2] in a review of urban EIRs. However they are lower than the results of Afrane *et al. *[9] who reported EIRs of 57 and 112.8 for UA and 1.2 and 18 for U in dry and rainy season respectively, in Kumasi, Ghana (their monthly figures were multiplied by 12 for comparison to the data presented herein).

## Conclusion

The results of this study show that urban malaria transmission is ongoing in Accra and that the EIR seems increased in urban areas where irrigated farming takes place. In addition, the urban setting seems to pose a number of unique challenges to malaria control that need further investigation, *e.g. *anophelines may be adapting to more polluted larval habitats, may be more exophilic than in rural settings thereby decreasing the efficacy of IRS and the intensive use of pesticides in urban agriculture is likely to increase the speed with which insecticide resistance develops.

## Competing interests

The authors declare that they have no competing interests.

## Authors' contributions

EK designed and carried out the survey and drafted the manuscript. MD and PMcC helped to design the study and finalized the manuscript. MW helped in the design of the study, provided backstopping during the fieldwork and provided critical comments on the manuscript. FA helped to design the study and helped to initiate the whole project. All authors (apart from FA) have read and approved the final manuscript. Sadly, Felix passed away in June 2005, but his invaluable contribution to the study merits his posthumous inclusion as co-author.
